# Development and validation of a bedside nomogram for predicting severe hospital-acquired pneumonia in traumatic brain injury patients based on early semi-quantitative chest CT assessment

**DOI:** 10.3389/fmed.2026.1926466

**Published:** 2026-10-30

**Authors:** Yanglingxi Wang, Weiduo Zhou, Qian Xiong, Dezhu Tang, Peng Chen, Yongbing Deng

**Affiliations:** 1 Department of Neurosurgery, Chongqing Emergency Medical Center, Chongqing University Central Hospital, School of Medicine, Chongqing University, Chongqing, China; 2 Department of Respiratory Medicine, Chongqing Emergency Medical Center, Chongqing University Central Hospital, School of Medicine, Chongqing University, Chongqing, China

**Keywords:** chest CT, nomogram, semi-quantitative scoring, severe hospital-acquired pneumonia, traumatic brain injury

## Abstract

**Introduction:**

Traumatic brain injury (TBI) often leads to severe hospital-acquired pneumonia (sHAP), which impairs recovery but remains difficult to predict early. We aimed to develop a bedside nomogram based on early chest CT features for individualized sHAP risk assessment in TBI patients.

**Methods:**

This retrospective study included TBI patients screened for sHAP. Early semi-quantitative chest CT assessment (ground-glass opacity [GGO] and consolidation scores) was combined with clinical and laboratory parameters. LASSO regression and subset selection were used for model development, followed by 10-fold cross-validation and independent validation.

**Results:**

An eight-variable model was established, including age, GCS score, mechanical ventilation, white blood cell count, lymphocyte count, hemoglobin, GGO score, and consolidation score. Multivariable analysis identified consolidation score (OR 1.20, 95% CI 1.03–1.39) and GGO score (OR 1.10, 95% CI 1.01–1.20) as independent risk factors. The nomogram showed robust discrimination with AUC of 0.880 (95% CI 0.851–0.908) in training and 0.871 (95% CI 0.824–0.918) in validation cohorts, with good calibration and clinical net benefit.

**Conclusions:**

This validated bedside nomogram enables early, individualized sHAP risk stratification in TBI management, supporting targeted clinical decision-making.

## Highlights

**Question:** Early identification of severe hospital-acquired pneumonia (sHAP) remains a diagnostic bottleneck in traumatic brain injury management, as traditional biomarkers often fail to distinguish neuro-inflammatory crosstalk from secondary pulmonary infections.

**Findings:** Integrating semi-quantitative chest CT scoring of ground-glass opacities and consolidation with early clinical indicators enables the construction of a high-performance nomogram, providing superior discriminative accuracy for sHAP risk.

**Clinical relevance:** This bedside tool offers a translatable framework for individualized risk stratification, potentially optimizing early intervention strategies and improving clinical trajectories for patients in neurocritical care settings.

## Introduction

Traumatic brain injury (TBI) is a leading cause of death and long-term disability worldwide, with an estimated annual incidence of 100–300 per 100,000 individuals (1). The burden of TBI is particularly pronounced among young adults aged 15–45 years, in whom it represents a major cause of death and severe functional impairment, thereby imposing a substantial socioeconomic burden on global public health systems (2, 3). Following TBI, injury to the central nervous system triggers a systemic inflammatory response that may subsequently evolve into a state of immunosuppression mediated by autonomic nervous system activation and neuroendocrine dysregulation (4). This immune dysfunction markedly compromises host defence against pathogens. In addition, brain injury–related reductions in consciousness and impaired airway protective reflexes further increase the risk of secretion aspiration and the development of hospital-acquired infections, particularly pneumonia (5).

Hospital-acquired pneumonia (HAP) is defined as pneumonia occurring ≥48 h after hospital admission that was neither present nor incubating at the time of admission (6). Among patients with TBI, HAP represents one of the most common infectious complications during hospitalization and remains highly prevalent worldwide. Previous studies have reported that the incidence of HAP in patients with TBI ranges from approximately 40% to 65%, particularly among those requiring intensive care or mechanical ventilation (7–9). The occurrence of HAP significantly prolongs the duration of mechanical ventilation, intensive care unit stay and overall hospitalization, while increasing disability and mortality. Severe hospital-acquired pneumonia (sHAP), a more advanced form of HAP, is associated with even poorer clinical outcomes and higher mortality rates. Therefore, early identification and prevention of sHAP are critical for improving the prognosis of patients with TBI.

Currently, risk assessment for pneumonia following TBI primarily relies on traditional clinical risk factors, inflammatory biomarkers and imaging evaluation (10–12). However, most existing prediction approaches emphasize conventional clinical variables and often lack disease-specific indicators, which may limit predictive accuracy. Laboratory markers such as blood cell counts, procalcitonin and C-reactive protein mainly reflect systemic inflammatory responses but lack specificity, as their levels can be influenced by non-infectious inflammation induced by traumatic brain injury itself (11). More recently, radiomics-based approaches have shown promise by extracting high-dimensional quantitative features from chest CT images to capture subtle structural alterations in lung parenchyma (12). Nevertheless, these methods require complex workflows involving standardized image acquisition, segmentation and computational feature extraction, which can be time-consuming and difficult to integrate into the rapid bedside decision-making processes typical of neurological critical care units (NCCU) (13).

Liu et al. previously proposed a simplified semi-quantitative CT scoring system to evaluate pulmonary involvement in patients with COVID-19, demonstrating promising prognostic value (14). Building upon this concept, the present study adapted and refined the CT scoring framework to better capture early pulmonary imaging characteristics in patients with traumatic brain injury managed in neurocritical care settings. We therefore aimed to develop and validate a practical bedside prediction model for severe hospital-acquired pneumonia (sHAP) by integrating early chest CT features with routinely available clinical variables. The proposed model is simple to implement and designed to facilitate rapid imaging-based risk stratification during the early phase of hospitalization, thereby helping clinicians identify patients at high risk of developing sHAP and enabling timely preventive and therapeutic interventions.

## Methods

### Patients

This retrospective cohort study included patients with traumatic brain injury (TBI) who were consecutively admitted to the Department of Neurosurgery at Chongqing Emergency Medical Center between January 2020 and December 2024. All clinical data were retrieved from the institutional electronic medical record system and the institutional traumatic brain injury electronic database.

The study protocol was reviewed and approved by the Ethics Committee of Chongqing Emergency Medical Center, and the requirement for informed consent was waived due to the retrospective nature of the study. All procedures were conducted in accordance with the principles of the Declaration of Helsinki. TBI was diagnosed based on clinical presentation, neurological examination, and radiological findings at admission, including computed tomography or magnetic resonance imaging. All patients received standardized neurocritical care management according to departmental protocols.

### Eligibility criteria

Patients were eligible for inclusion if they met the following criteria: (1) a primary diagnosis of traumatic brain injury, with or without concomitant systemic injuries (e.g., thoracic, abdominal, or limb injuries); (2) age between 18 and 75 years; (3) hospital admission within 6 h after injury; (4) absence of clinical or radiological evidence of community-acquired pneumonia at admission and no development of pneumonia within the first 48 h of hospitalization; (5) complete and accessible medical records throughout the hospitalization period.

Patients were excluded if they had: (1) pre-existing chronic pulmonary diseases, including chronic obstructive pulmonary disease, asthma, interstitial lung disease, pulmonary hypertension, or chronic pulmonary embolism; (2) chronic inflammatory or autoimmune diseases, such as rheumatoid arthritis, Crohn's disease, ulcerative colitis, or other systemic autoimmune disorders; (3) long-term use of immunosuppressive agents or systemic corticosteroids; (4) documented chronic liver or renal disease; (5) a history of acute or chronic heart failure; (6) a prior diagnosis of malignancy; (7) a recent history of major surgery unrelated to the index trauma; (8) an estimated life expectancy of less than two weeks at the time of admission.

A total of 827 patients with TBI were initially screened. After applying the predefined inclusion and exclusion criteria, 46 patients were excluded, resulting in a final analytic cohort of 781 patients (in Figure 1).

**Figure 1:**
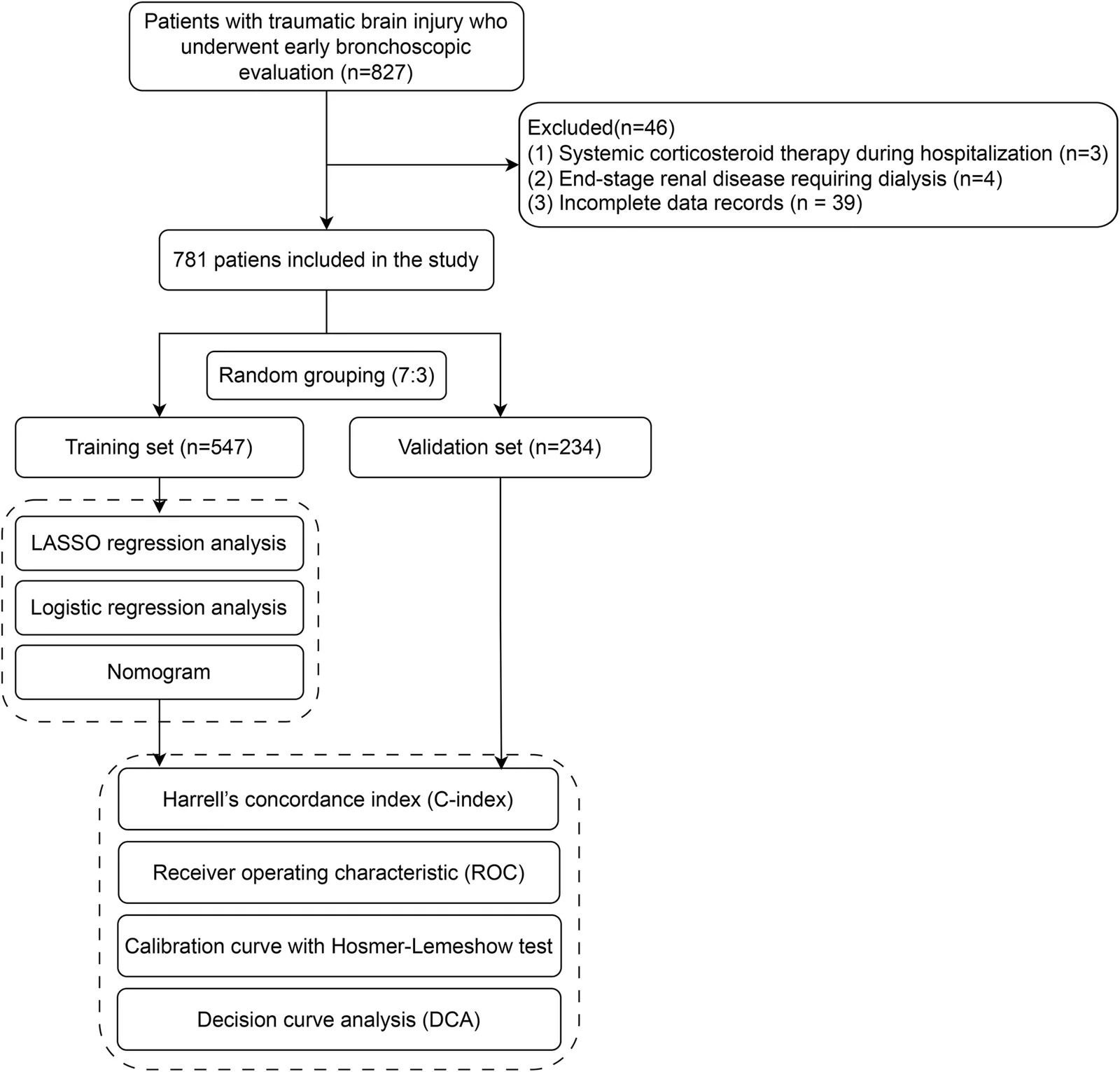
Flow chart of inclusion and exclusion criteria for research subjects. A total of 827 patients with traumatic brain injury (TBI) who underwent early bronchoscopic evaluation were initially screened. Following the application of predefined inclusion and exclusion criteria, the final study cohort was established and subsequently randomized into training and validation sets at a 7:3 ratio for model development and performance evaluation.

### Data collection and potential predictors

Clinical data were extracted from the institutional electronic medical record system and the traumatic brain injury electronic database using a predefined data dictionary developed through literature review and expert consensus in neurotrauma and respiratory critical care. Unless otherwise specified, candidate predictors were collected within the first 48 h after admission and prior to the diagnosis of hospital-acquired pneumonia.

Baseline demographic characteristics included age and sex. Injury severity and neurological status at admission were assessed using the Injury Severity Score (ISS) and the Glasgow Coma Scale (GCS). Pre-existing chronic comorbidities were recorded based on documented medical history.

Respiratory- and thoracic-related variables included the requirement for mechanical ventilation. Chest computed tomography findings were systematically reviewed and included rib fractures, pneumothorax, pleural effusion, hemothorax, patchy consolidations, lower-lobe-confined consolidation, radiographic signs of chronic lung disease, pulmonary solid nodules, ground-glass opacities (GGO), cardiomegaly, pericardial effusion, and coronary atherosclerosis. Cardiac function was assessed using left ventricular ejection fraction derived from transthoracic echocardiography.

Laboratory parameters reflecting hematological status, inflammatory response, and tissue perfusion included white blood cell count, hemoglobin, hematocrit, platelet count, absolute neutrophil, monocyte, and lymphocyte counts, lactate, procalcitonin, and interleukin-6.

In addition, detailed classification criteria and quantitative definitions for trauma-related and chest imaging variables are provided in the footnotes of Tables 1, 2 to facilitate interpretation.

**Table 1:** Baseline characteristics of participants in training and validation set.

Variables	Total (*n* = 781)	Training set (*n* = 547) M (Q25, Q75)/N (%)	Validation set (*n* = 234) M (Q25, Q75)/N (%)	*P* value
Age, Mean ± SD	55.3 ± 17.5	56.2 ± 17.1	53.2 ± 18.3	0.028
Sex, *n* (%)				0.531
Male	566 (72.5)	400 (73.1)	166 (70.9)	
Female	215 (27.5)	147 (26.9)	68 (29.1)	
ISS, Median (IQR)^a^	16.0 (10.0, 25.0)	16.0 (9.0, 25.0)	18.0 (12.0, 27.0)	0.086
Chronic comorbidities, *n* (%)				0.098
No	507 (64.9)	345 (63.1)	162 (69.2)	
Yes	274 (35.1)	202 (36.9)	72 (30.8)	
GGO score, Median (IQR)	3.0 (2.0, 5.0)	3.0 (2.0, 5.0)	3.0 (1.2, 4.0)	0.959
Left rib fracture, *n* (%)				0.924
No	589 (75.4)	412 (75.3)	177 (75.6)	
Yes	192 (24.6)	135 (24.7)	57 (24.4)	
Right rib fracture, *n* (%)				0.208
No	605 (77.5)	417 (76.2)	188 (80.3)	
Yes	176 (22.5)	130 (23.8)	46 (19.7)	
Left-sided pneumothorax, Mean ± SD	0.5 ± 3.6	0.5 ± 3.5	0.6 ± 3.7	0.897
Right-sided pneumothorax, Mean ± SD	0.4 ± 3.2	0.4 ± 3.8	0.3 ± 1.5	0.619
Pleural effusion, *n* (%)^b^				0.8
None or minimal	609 (78.0)	428 (78.2)	181 (77.4)	
Moderate	153 (19.6)	107 (19.6)	46 (19.7)	
Severe	19 (2.4)	12 (2.2)	7 (3)	
Hemothorax, *n* (%)^c^				0.434
None or minimal	741 (94.9)	518 (94.7)	223 (95.3)	
Moderate	31 (4.0)	24 (4.4)	7 (3)	
Severe	9 (1.2)	5 (0.9)	4 (1.7)	
Consolidation score, Median (IQR)	2.0 (0.0, 4.0)	2.0 (0.0, 4.0)	2.0 (0.2, 4.0)	0.388
Lower-lobe-confined consolidation^d^, *n* (%)				0.703
No consolidation	216 (27.7)	156 (28.5)	60 (25.6)	
Lower-lobe confined	431 (55.2)	299 (54.7)	132 (56.4)	
Beyond lower lobes	134 (17.2)	92 (16.8)	42 (17.9)	
Radiographic signs of chronic lung disease, *n* (%)^e^				0.921
No	526 (67.3)	369 (67.5)	157 (67.1)	
Yes	255 (32.7)	178 (32.5)	77 (32.9)	
Pulmonary solid nodule, *n* (%)				0.761
No	417 (53.4)	294 (53.7)	123 (52.6)	
Yes	364 (46.6)	253 (46.3)	111 (47.4)	
Cardiomegaly, *n* (%)				0.927
No	569 (72.9)	398 (72.8)	171 (73.1)	
Yes	212 (27.1)	149 (27.2)	63 (26.9)	
Pericardial effusion, *n* (%)				0.575
No	727 (93.1)	511 (93.4)	216 (92.3)	
Yes	54 (6.9)	36 (6.6)	18 (7.7)	
Coronary atherosclerosis, *n* (%)				0.574
No	442 (56.6)	306 (55.9)	136 (58.1)	
Yes	339 (43.4)	241 (44.1)	98 (41.9)	
LVEF^f^, Mean ± SD	62.9 ± 5.7	62.8 ± 5.8	63.1 ± 5.5	0.534
Mechanical ventilation, *n* (%)				0.076
No	546 (69.9)	372 (68)	174 (74.4)	
Yes	235 (30.1)	175 (32)	60 (25.6)	
GCS score^g^, Mean ± SD	11.1 ± 3.6	11.1 ± 3.7	11.3 ± 3.5	0.445
sHAP, *n* (%)				0.065
No	534 (68.4)	363 (66.4)	171 (73.1)	
Yes	247 (31.6)	184 (33.6)	63 (26.9)	
Laboratory values				
WBC (×109/L)	11.3 ± 4.8	11.3 ± 4.8	11.3 ± 4.6	0.964
HGB (g/L)	114.8 ± 23.2	114.5 ± 23.4	115.3 ± 22.7	0.663
HCT (%)	33.8 ± 9.2	33.6 ± 9.6	34.3 ± 8.1	0.319
PLT (×109/L)	168.3 ± 67.1	167.9 ± 67.2	169.4 ± 66.9	0.771
Neu# (×109/L)	8.9 (6.5, 11.9)	8.9 (6.6, 11.9)	8.7 (6.3, 11.9)	0.693
Mon# (×109/L)	0.6 (0.4, 0.8)	0.6 (0.4, 0.8)	0.7 (0.5, 0.9)	0.155
Lym# (×109/L)	0.9 (0.6, 1.2)	0.9 (0.6, 1.2)	0.9 (0.6, 1.3)	0.519
LAC (mmol/L)	2.5 (2.0, 3.5)	2.6 (2.0, 3.5)	2.5 (2.0, 3.3)	0.135
PCT (%)	0.2 (0.1, 0.8)	0.2 (0.1, 0.8)	0.3 (0.1, 1.0)	0.573
IL−6(pg/ml)	43.6 (12.9, 127.2)	44.9 (13.7, 131.2)	42.9 (10.8, 110.3)	0.317

sHAP, severe hospital-acquired pneumonia; ISS, Injury severity score; GCS, glasgow coma scale; LVEF, Left ventricular ejection fraction; WBC, white blood count; HGB, hemoglobin; HCT, hematocrit; PLT, platelet; Neu, neutrophil; Mon, monocyte; Lym, lymphocyte; LAC, blood lactic acid.

a ISS,Injury severity score: The Injury Severity Score (ISS) is a scoring system used to assess the overall severity of injuries in trauma patients. The ISS is based on the anatomical regions of injury, dividing the body into six areas: head/neck, face, chest, abdomen or pelvis, extremities or pelvic girdle, and external. Each region is scored according to the severity of the injury, and the ISS is calculated as the sum of the squares of the three most severely injured regions. The total ISS ranges from 1 to 75.

b Pleural effusion: Pleural effusion was identified as low-attenuation fluid collections within the pleural space, characterized by a CT density typically ranging from 0 to 20 Hounsfield Units (HU). Grading was semi-quantitatively performed based on the maximum anteroposterior (AP) depth measured on supine chest CT: none or minimal, defined as an AP depth <3 cm, representing limited serous or inflammatory exudate with negligible impact on pulmonary ventilation; moderate, defined as an AP depth between 3 and 10 cm, associated with partial compression of the adjacent lung parenchyma; and severe, defined as an AP depth exceeding 10 cm, which frequently leads to substantial compressive atelectasis or mediastinal shift.

c Hemothorax: Hemothorax was distinguished from simple effusion by the presence of high-attenuation pleural fluid, exhibiting a CT density typically between 35 and 70 HU, indicative of acute intrapleural hemorrhage. Given the elevated risks of hemorrhagic shock and clotted fibrothorax, a stricter grading scale was applied: none or minimal, defined as an AP depth <2 cm, usually localized to the costophrenic angle without significant hemodynamic compromise; moderate, defined as an AP depth between 2 and 5 cm, generally warranting clinical drainage; and severe, defined as an AP depth exceeding 5 cm, often corresponding to massive hemothorax and characterized by acute lung collapse with a high risk of circulatory failure.

d Lower-lobe-confined consolidation: Lung consolidation was identified on CT as high-attenuation areas obscuring underlying vascular structures, frequently accompanied by air bronchograms, and was categorized into three grades based on its spatial involvement: none, characterized by the absence of identifiable consolidative opacities; confined to bilateral lower lobes, where consolidative lesions were restricted to the inferior lobes of both lungs, a pattern typically reflecting dependent lung changes or localized aspiration common in bedridden patients; and not confined to bilateral lower lobes, where consolidative involvement extended to the middle or upper lobes or presented as diffuse infiltration, indicating more severe parenchymal insult such as multilobar pneumonia or acute respiratory distress syndrome (ARDS). This classification was designed to distinguish gravity-dependent physiological alterations from extensive parenchymal damage and its associated risk of significant oxygenation impairment.

e Radiographic signs of chronic lung disease: Chronic lung disease was identified on chest CT as structural alterations reflecting long-term parenchymal remodeling or airway damage. These radiographic signatures included, but were not limited to: emphysema, characterized by areas of low attenuation without discrete walls; pulmonary fibrosis, manifested as reticular opacities, honeycombing, or traction bronchiectasis; and chronic airway disease, indicated by bronchial wall thickening or irreversible bronchiectasis. This assessment was designed to identify patients with pre-existing lung impairment, a demographic at increased risk for secondary complications following traumatic or critical insult.

f LVEF: Left ventricular ejection fraction was quantified via point-of-care ultrasound (POCUS) or standard echocardiography, utilizing the modified Simpson's method (biplane method of disks). LVEF is defined as the percentage of blood ejected from the left ventricle during each contraction relative to its end-diastolic volume, serving as the gold-standard metric for assessing global ventricular systolic function. A reduced LVEF indicates impaired myocardial contractility or pre-existing cardiac dysfunction, which may predispose patients to hemodynamic instability, inadequate organ perfusion, and diminished tolerance to fluid resuscitation following critical insult.

g GCS, glasgow coma scale: The Glasgow Coma Scale (GCS) is a standard tool for assessing the level of consciousness. It evaluates eye-opening, verbal, and motor responses, producing a total score ranging from 3 to 15. The GCS is widely used for the initial assessment and ongoing monitoring of traumatic brain injury patients, with lower scores indicating more severe impairment of consciousness.

**Table 2:** Baseline characteristics of participants.

Variables	Total (*n* = 781)	sHAP		*P* value
		No (*n* = 534)	Yes (*n* = 247)	
Age, Mean ± SD	55.3 ± 17.5	53.3 ± 17.7	59.5 ± 16.2	<0.001
Sex, *n* (%)				0.085
Male	566 (72.5)	377 (70.6)	189 (76.5)	
Female	215 (27.5)	157 (29.4)	58 (23.5)	
ISS, Median (IQR)^a^	16.0 (10.0, 25.0)	16.0 (9.0, 23.0)	25.0 (16.0, 31.0)	<0.001
Chronic comorbidities, *n* (%)				0.306
No	507 (64.9)	353 (66.1)	154 (62.3)	
Yes	274 (35.1)	181 (33.9)	93 (37.7)	
GGO score, Median (IQR)	3.0 (2.0, 5.0)	2.0 (1.0, 4.0)	4.0 (2.0, 5.0)	<0.001
Left rib fracture, *n* (%)				<0.001
No	589 (75.4)	429 (80.3)	160 (64.8)	
Yes	192 (24.6)	105 (19.7)	87 (35.2)	
Right rib fracture, *n* (%)				<0.001
No	605 (77.5)	436 (81.6)	169 (68.4)	
Yes	176 (22.5)	98 (18.4)	78 (31.6)	
Left-sided pneumothorax, Mean ± SD	0.5 ± 3.6	0.2 ± 1.4	1.2 ± 6.0	<0.001
Right-sided pneumothorax, Mean ± SD	0.4 ± 3.2	0.1 ± 0.8	1.1 ± 5.6	<0.001
Pleural effusion, *n* (%)^b^				<0.001
None or minimal	609 (78.0)	447 (83.7)	162 (65.6)	
Moderate	153 (19.6)	81 (15.2)	72 (29.1)	
Severe	19 (2.4)	6 (1.1)	13 (5.3)	
Hemothorax, *n* (%)^c^				<0.001
None or minimal	741 (94.9)	519 (97.2)	222 (89.9)	
Moderate	31 (4.0)	11 (2.1)	20 (8.1)	
Severe	9 (1.2)	4 (0.7)	5 (2)	
Consolidation score, Median (IQR)	2.0 (0.0, 4.0)	2.0 (0.0, 3.0)	4.0 (2.0, 6.0)	<0.001
Lower-lobe-confined consolidation^d^, *n* (%)				<0.001
No consolidation	216 (27.66)	180 (33.7)	36 (14.6)	
Lower-lobe confined	431 (55.18)	288 (53.9)	143 (57.9)	
Beyond lower lobes	134 (17.16)	66 (12.4)	68 (27.5)	
Radiographic signs of chronic lung disease, *n* (%)^e^				0.446
No	526 (67.3)	355 (66.5)	171 (69.2)	
Yes	255 (32.7)	179 (33.5)	76 (30.8)	
Pulmonary solid nodule, *n* (%)				0.345
No	417 (53.4)	279 (52.2)	138 (55.9)	
Yes	364 (46.6)	255 (47.8)	109 (44.1)	
Cardiomegaly, *n* (%)				<0.001
No	569 (72.9)	413 (77.3)	156 (63.2)	
Yes	212 (27.1)	121 (22.7)	91 (36.8)	
Pericardial effusion, *n* (%)				0.135
No	727 (93.1)	502 (94)	225 (91.1)	
Yes	54 (6.9)	32 (6)	22 (8.9)	
Coronary atherosclerosis, *n* (%)				0.002
No	442 (56.6)	322 (60.3)	120 (48.6)	
Yes	339 (43.4)	212 (39.7)	127 (51.4)	
LVEF^f^, Mean ± SD	62.9 ± 5.7	62.9 ± 5.7	62.9 ± 5.7	0.868
Mechanical ventilation, *n* (%)				<0.001
No	546 (69.9)	452 (84.6)	94 (38.1)	
Yes	235 (30.1)	82 (15.4)	153 (61.9)	
GCS^g^ score, Mean ± SD	11.1 ± 3.6	12.2 ± 3.1	8.8 ± 3.5	<0.001
Laboratory values				
WBC (×109/L)	11.3 ± 4.8	10.3 ± 3.9	13.5 ± 5.7	<0.001
HGB (g/L)	114.8 ± 23.2	118.3 ± 22.3	107.0 ± 23.1	<0.001
HCT (%)	33.8 ± 9.2	34.7 ± 9.3	31.8 ± 8.4	<0.001
PLT (×109/L)	168.3 ± 67.1	178.6 ± 67.6	146.0 ± 60.4	<0.001
Neu# (×109/L)	8.9 (6.5, 11.9)	8.0 (5.9, 10.6)	11.1 (8.6, 14.0)	<0.001
Mon# (×109/L)	0.6 (0.4, 0.8)	0.6 (0.4, 0.8)	0.7 (0.5, 0.9)	0.042
Lym# (×109/L)	0.9 (0.6, 1.2)	0.9 (0.6, 1.3)	0.8 (0.5, 1.0)	<0.001
LAC (mmol/L)	2.5 (2.0, 3.5)	2.4 (1.9, 3.3)	3.0 (2.3, 4.0)	<0.001
PCT (%)	0.2 (0.1, 0.8)	0.1 (0.0, 0.5)	0.6 (0.2, 1.8)	<0.001
IL−6(pg/ml)	43.6 (12.9, 127.2)	27.5 (9.0, 77.4)	102.5 (46.1, 226.6)	<0.001

sHAP, severe hospital-acquired pneumonia; ISS, Injury severity score; GCS, glasgow coma scale; LVEF, Left ventricular ejection fraction; WBC, white blood count; HGB, hemoglobin; HCT, hematocrit; PLT, platelet; Neu, neutrophil; Mon, monocyte; Lym, lymphocyte; LAC, blood lactic acid.

a ISS,Injury severity score: The Injury Severity Score (ISS) is a scoring system used to assess the overall severity of injuries in trauma patients. The ISS is based on the anatomical regions of injury, dividing the body into six areas: head/bck, face, chest, abdomen or pelvis, extremities or pelvic girdle, and external. Each region is scored according to the severity of the injury, and the ISS is calculated as the sum of the squares of the three most severely injured regions. The total ISS ranges from 1 to 75.

b Pleural effusion: Pleural effusion was identified as low-attenuation fluid collections within the pleural space, characterized by a CT density typically ranging from 0 to 20 Hounsfield Units (HU). Grading was semi-quantitatively performed based on the maximum anteroposterior (AP) depth measured on supine cbhest CT: none or minimal, defined as an AP depth <3 cm, representing limited serous or inflammatory exudate with negligible impact on pulmonary ventilation; moderate, defined as an AP depth between 3 and 10 cm, associated with partial compression of the adjacent lung parenchyma; and severe, defined as an AP depth exceeding 10 cm, which frequently leads to substantial compressive atelectasis or mediastinal shift.

c Hemothorax: Hemothorax was distinguished from simple effusion by the presence of high-attenuation pleural fluid, exhibiting a CT density typically between 35 and 70 HU, indicative of acute intrapleural hemorrhage. Given the elevated risks of hemorrhagic shock and clotted fibrothorax, a stricter grading scale was applied: none or minimal, defined as an AP depth <2 cm, usually localized to the costophrenic angle without significant hemodynamic compromise; moderate, defined as an AP depth between 2 and 5 cm, generally warranting clinical drainage; and severe, defined as an AP depth exceeding 5 cm, often corresponding to massive hemothorax and characterized by acute lung collapse with a high risk of circulatory failure.

d Lower-lobe-confined consolidation: Lung consolidation was identified on CT as high-attenuation areas obscuring underlying vascular structures, frequently accompanied by air bronchograms, and was categorized into three grades based on its spatial involvement: none, characterized by the absence of identifiable consolidative opacities; confined to bilateral lower lobes, where consolidative lesions were restricted to the inferior lobes of both lungs, a pattern typically reflecting dependent lung changes or localized aspiration common in bedridden patients; and not confined to bilateral lower lobes, where consolidative involvement extended to the middle or upper lobes or presented as diffuse infiltration, indicating more severe parenchymal insult such as multilobar pneumonia or acute respiratory distress syndrome (ARDS). This classification was designed to distinguish gravity-dependent physiological alterations from extensive parenchymal damage and its associated risk of significant oxygenation impairment.

e Radiographic signs of chronic lung disease: Chronic lung disease was identified on chest CT as structural alterations reflecting long-term parenchymal remodeling or airway damage. These radiographic signatures included, but were not limited to: emphysema, characterized by areas of low attenuation without discrete walls; pulmonary fibrosis, manifested as reticular opacities, honeycombing, or traction bronchiectasis; and chronic airway disease, indicated by bronchial wall thickening or irreversible bronchiectasis. This assessment was designed to identify patients with pre-existing lung impairment, a demographic at increased risk for secondary complications following traumatic or critical insult.

f LVEF: Left ventricular ejection fraction was quantified via point-of-care ultrasound (POCUS) or standard echocardiography, utilizing the modified Simpson's method (biplane method of disks). LVEF is defined as the percentage of blood ejected from the left ventricle during each contraction relative to its end-diastolic volume, serving as the gold-standard metric for assessing global ventricular systolic function. A reduced LVEF indicates impaired myocardial contractility or pre-existing cardiac dysfunction, which may predispose patients to hemodynamic instability, inadequate organ perfusion, and diminished tolerance to fluid resuscitation following critical insult.

g GCS,glasgow coma scale: The Glasgow Coma Scale (GCS) is a standard tool for assessing the level of consciousness. It evaluates eye-opening, verbal, and motor responses, producing a total score ranging from 3 to 15. The GCS is widely used for the initial assessment and ongoing monitoring of traumatic brain injury patients, with lower scores indicating more severe impairment of consciousness.

### Chest CT acquisition and image analysis

All chest CT scans were obtained within 48 h of hospital admission using a 256-slice, 512-detector-row CT system (Revolution CT, GE Healthcare, WI, USA), covering the entire lung field. Images were independently reviewed by two experienced respiratory physicians who were blinded to clinical outcomes. When discrepancies occurred in the presence, extent, or lobar distribution of GGO or consolidation, the two reviewers re-evaluated the corresponding CT images together and reached a final score through consensus discussion according to the predefined scoring criteria. No formal interobserver reliability analysis, such as kappa statistics, was performed in this retrospective study.

For early-stage chest CT scans, two imaging features were evaluated: ground-glass opacities (GGO) and patchy consolidations. GGO was defined as a mild increase in pulmonary parenchymal attenuation with preservation of bronchial and vascular margins, whereas consolidations were characterized by a marked increase in attenuation with obscuration of these structures, reflecting complete replacement of alveolar air by fluid, inflammatory cells, or other materials (15).

In the acute trauma setting, two blinded reviewers assessed possible pulmonary contusion or intraparenchymal hemorrhage according to attenuation, morphology, lobar distribution, relationship to adjacent thoracic injury (e.g., rib fracture or hemothorax), and pleural findings; no Hounsfield-unit threshold was applied. Because contusion, hemorrhage, edema, aspiration-related injury, and early inflammation may overlap on CT, these lesions were not regarded as pneumonia at admission and were not excluded from scoring. They were assigned to the GGO or consolidation category by attenuation pattern and included in the corresponding total score (range, 0–25). The scores were used as semi-quantitative descriptors of early parenchymal involvement for subsequent sHAP risk prediction.

The chest CT images were anatomically segmented into five lobar regions according to standard bronchial anatomy and interlobar fissures: the right lung into the upper, middle, and lower lobes, and the left lung into the upper and lower lobes (15).

For each lung lobe, the extent of GGO and consolidations were assessed separately using a semi-quantitative scoring system: 0 (0%), 1 (<5%), 2 (5%–25%), 3 (26%–50%), 4 (51%–75%), and 5 (>75%) of lobar involvement. The scores from the five lobes were summed to generate two composite indicators: the GGO score and the consolidation score, each ranging from 0 to 25. Higher scores indicate a greater extent of pulmonary involvement (in Supplementary Figure S1).

### Definition of severe hospital-acquired pneumonia

Severe hospital-acquired pneumonia (sHAP) was defined as pneumonia occurring at least 48 h after hospital admission in patients with traumatic brain injury who had no clinical or radiological evidence of pulmonary infection at admission or within the first 48 h of hospitalization. The diagnosis and severity classification of sHAP were determined in accordance with the 2016 Infectious Diseases Society of America/American Thoracic Society (IDSA/ATS) guidelines (16).

Specifically, sHAP was diagnosed when patients met at least one major criterion—requiring vasopressor support for septic shock—or at least three minor criteria, including: (a) respiratory rate >30 breaths·min^−1^; (b) PaO_2_/FiO_2_ ≤ 250; (c) multilobar pulmonary infiltrates on imaging; (d) new-onset altered mental status; (e) blood urea nitrogen ≥7.1 mmol·L^−1^; (f) leukopenia (white blood cell count <4 × 10^9^·L^−1^); (g) thrombocytopenia (platelet count <100 × 10^9^·L^−1^); (h) core temperature <36°C; or (i) hypotension requiring aggressive fluid resuscitation.

Thrombocytopenia was used only as one of the IDSA/ATS minor criteria and was never sufficient by itself to classify sHAP. When a platelet decline was recorded at the time of severity assessment, the chart was reviewed for concurrent infection, bleeding, trauma-related coagulopathy, drug exposure, and the timing of pharmacological thromboprophylaxis. In this TBI cohort, drug prophylaxis was started only after individualized assessment of bleeding risk and neurological stability. HIT assays and 4Ts scores were not obtained systematically; therefore heparin-induced thrombocytopenia could not be formally excluded in every case and was not treated as a separate diagnostic category.

Patients who did not meet the above criteria, including those without pneumonia or with non-severe hospital-acquired pneumonia, were categorized into the non-sHAP group.

Standardized preventive measures were implemented for all patients in accordance with the Chinese Expert Consensus on Infection Management in Neurosurgical Critically Ill Patients.

These interventions encompassed a multi-modal care bundle, including head-of-bed elevation (30–45°), subglottic secretion drainage, oral decontamination with chlorhexidine, and daily spontaneous awakening trials (SATs) to minimize mechanical ventilation duration (17).

### Logistic regression analysis and nomogram model development

A total of 781 eligible patients were randomly allocated to a training cohort and a validation cohort at a ratio of 7:3. Baseline demographic and clinical characteristics were compared between the two cohorts to ensure comparability prior to model development.

To identify candidate predictors of severe hospital-acquired pneumonia, least absolute shrinkage and selection operator (LASSO) regression was first applied in the training cohort to reduce dimensionality and mitigate potential multicollinearity among candidate variables. Variables with non-zero coefficients were retained as candidate predictors for subsequent modeling.

To further guide model complexity and ensure model parsimony, best subset selection based on the Bayesian information criterion (BIC) was additionally performed. This procedure was used to determine the optimal number of predictors to be retained in the final model rather than to independently select variables.

Candidate predictors identified by LASSO were subsequently entered into a multivariable logistic regression model. To assess the adequacy of model events relative to the number of predictors, the events-per-variable (EPV) value was calculated using the number of sHAP events in the training cohort. Multicollinearity among candidate predictors was evaluated using the variance inflation factor (VIF), with a VIF value <5 considered acceptable for inclusion in the multivariable logistic regression model. Predictors that were not statistically significant were sequentially removed while ensuring that the final model size remained consistent with the number of predictors suggested by the BIC-based subset selection.

The final set of predictors was then used to construct a nomogram for estimating the probability of severe hospital-acquired pneumonia.

### Apparent performance of the nomogram

The predictive performance of the constructed nomogram was evaluated from both discrimination and calibration perspectives. Model discrimination was quantified using Harrell's concordance index (C-index) and the area under the receiver operating characteristic curve (AUC), which together reflect the model's ability to distinguish between patients who developed severe hospital-acquired pneumonia and those who did not. A higher C-index and an AUC exceeding 0.80 were interpreted as indicators of strong discriminative capability. Calibration was assessed by examining the agreement between predicted probabilities and observed outcomes. Calibration plots were generated for both the training and validation cohorts, accompanied by the Hosmer-Lemeshow goodness-of-fit test to evaluate the consistency of model predictions. To further determine the clinical utility of the prediction model, decision curve analysis (DCA) was performed across a range of threshold probabilities, quantifying the net benefit of applying the nomogram in clinical decision-making. Both discrimination and calibration assessments were internally validated using 1,000 ordinary bootstrap resamples to reduce overfitting and assess the robustness of model performance. Percentile confidence intervals were obtained; BCa intervals were not applied.

### Statistical analysis

The distribution of continuous variables was assessed using the Shapiro–Wilk test in combination with visual inspection of histograms and Q-Q plots. Variables showing an approximately symmetric distribution with no marked deviation on graphical assessment were summarized as mean ± standard deviation, whereas skewed variables were reported as median with interquartile ranges. Comparisons between groups were performed using the independent samples t-test for normally distributed variables, and the Welch *t*-test was applied when heterogeneity of variance was detected. For non-normally distributed data, the Mann–Whitney *U*-test was used. Categorical variables were expressed as counts and percentages, and group differences were evaluated using the chi-square test or Fisher's exact test as appropriate. All statistical analyses were conducted using R software version 4.0.2 (The R Project for Statistical Computing, https://www.r-project.org) and Free Statistics version 2.1, which is based on the R statistical environment. Multivariable logistic regression and nomogram construction were performed using R-based procedures, including functions from the rms package. ROC, calibration, and decision curve analyses were conducted through the Free Statistics interface. A two-tailed *P* value < 0.05 was considered statistically significant.

## Results

### Patient characteristics

A total of 781 patients met the eligibility criteria and were included in the final analysis. The cohort was randomly divided into a training set (*n* = 547) and a validation set (*n* = 234). During hospitalization, 247 patients developed sHAP, corresponding to an overall incidence of 31.6% in the study population. The proportion of patients with sHAP did not differ significantly between the training and validation cohorts, indicating good comparability between the two datasets. The baseline demographic, clinical, radiological and laboratory characteristics of the study population are summarized in Tables 1, 2.

### Predictor selection

Given the large number of candidate variables included in the analysis, a multistep feature selection strategy was adopted. Initially, LASSO regression was performed in the training cohort to reduce dimensionality and address potential multicollinearity among candidate predictors. This procedure identified ten variables with non-zero coefficients for further evaluation (in Supplementary Figures S2A,B).

To further evaluate model parsimony and guide the appropriate model complexity, best subset selection based on the Bayesian information criterion (BIC) was additionally performed. This analysis indicated that a model including approximately eight predictors provided the optimal balance between model fit and parsimony.

The ten variables identified by LASSO were subsequently entered into a multivariable logistic regression model. During model refinement, predictors that did not show statistically significant associations or contributed minimally to model performance were sequentially removed while maintaining consistency with the model size suggested by the BIC analysis. Ultimately, eight predictors were retained in the final model. In the training cohort, 184 patients developed sHAP, yielding an EPV of 23.0 for the final eight-variable model and indicating an adequate event-to-predictor ratio for multivariable logistic regression.

As shown in Figure 2, increasing age (OR = 1.02, 95% CI 1.00–1.04, *P* = 0.032), lower GCS score (OR = 0.84, 95% CI 0.78–0.91, *P* < 0.001), mechanical ventilation (OR = 3.94, 95% CI 2.14–7.28, *P* < 0.001), GGO score (OR = 1.10, 95% CI 1.01–1.20, *P* = 0.038), white blood cell count (OR = 1.23, 95% CI 1.14–1.33, *P* < 0.001), and consolidation score (OR = 1.20, 95% CI 1.03–1.39, *P* = 0.021) were identified as risk factors. In contrast, higher lymphocyte count (OR = 0.59, 95% CI 0.40–0.86, *P* = 0.007) and hemoglobin level (OR = 0.99, 95% CI 0.97–1.00, *P* = 0.042) were associated with a reduced likelihood of sHAP.

**Figure 2:**
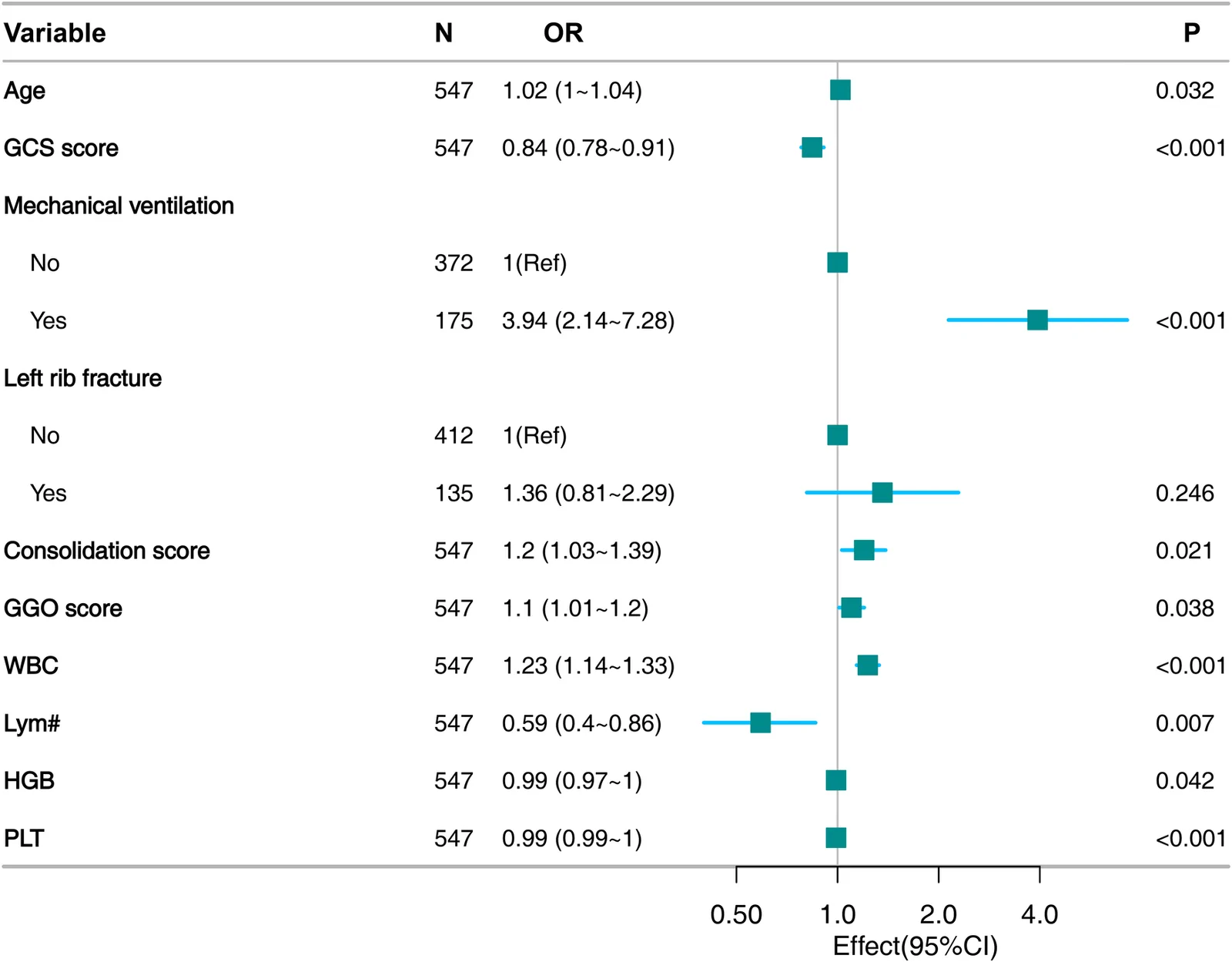
Forest plot of candidate predictors evaluated in the multivariable logistic regression analysis for sHAP in patients with TBI. Advanced age, mechanical ventilation, higher WBC count, GGO score, and consolidation score were associated with increased sHAP risk, whereas higher GCS score, lymphocyte count, and hemoglobin level were associated with reduced risk. For example, each 1-year increase in age corresponded to an approximately 2% increase in the odds of sHAP, each 1-point increase in GGO score corresponded to an approximately 10% increase, and each 1-point increase in consolidation score corresponded to an approximately 20% increase. Mechanical ventilation was associated with approximately 3.94-fold higher odds of sHAP compared with no mechanical ventilation. The final nomogram was constructed using the retained predictors after considering statistical significance, model parsimony, and clinical interpretability.

These eight predictors were incorporated into the final model and subsequently used to construct the nomogram in the training cohort, which was later validated in the independent validation cohort.

### Model development

An individualized prediction model for severe hospital-acquired pneumonia was constructed by incorporating the eight independent predictors identified in the multivariable logistic regression analysis. These predictors were integrated into a nomogram designed to estimate the probability of sHAP for individual patients. The nomogram provides a graphical representation of the model, enabling clinicians to derive a predicted risk by summing the point values assigned to each variable. The completed nomogram based on the training cohort is presented in Figure 3.

**Figure 3:**
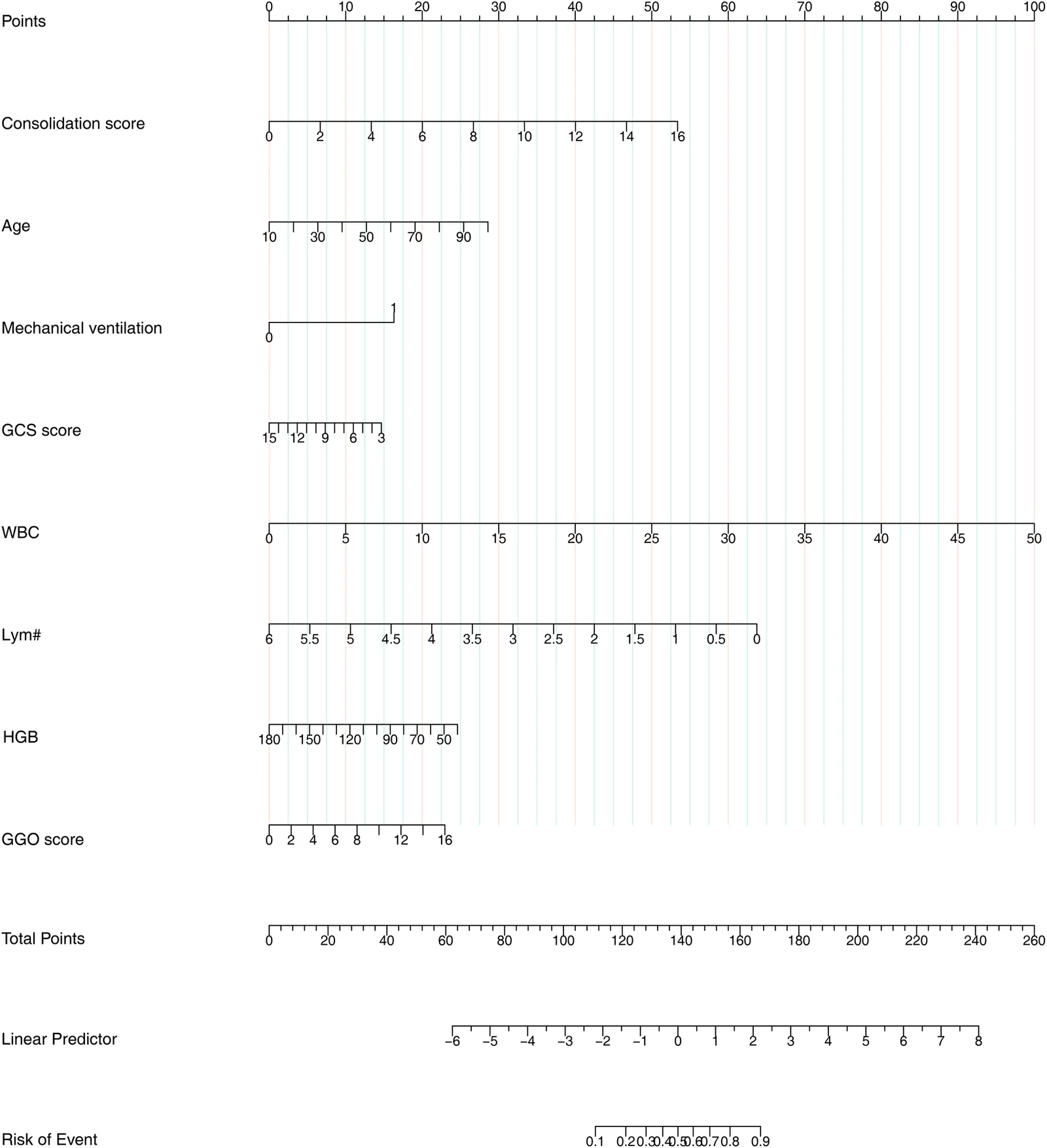
Nomogram for individualized sHAP risk prediction in TBI patients. The nomogram, developed from the training cohort, integrates eight independent predictors—including clinical, radiological, and laboratory parameters—to provide individualized risk estimates for sHAP. By summing the scores of each variable, the model yields a graphical probability of sHAP for clinical decision-making.

### Model discrimination

The discrimination performance of the predictive model was first assessed using the area under the receiver operating characteristic curve (AUC). In the training cohort, the nomogram demonstrated strong discriminative ability, yielding an AUC of 0.880 (95% CI: 0.851–0.908). Consistent performance was observed in the validation cohort, where the AUC remained high at 0.871 (95% CI: 0.824–0.918). These findings indicate that the model maintained robust classification capability across both datasets (in Figures 4A,B).

**Figure 4:**
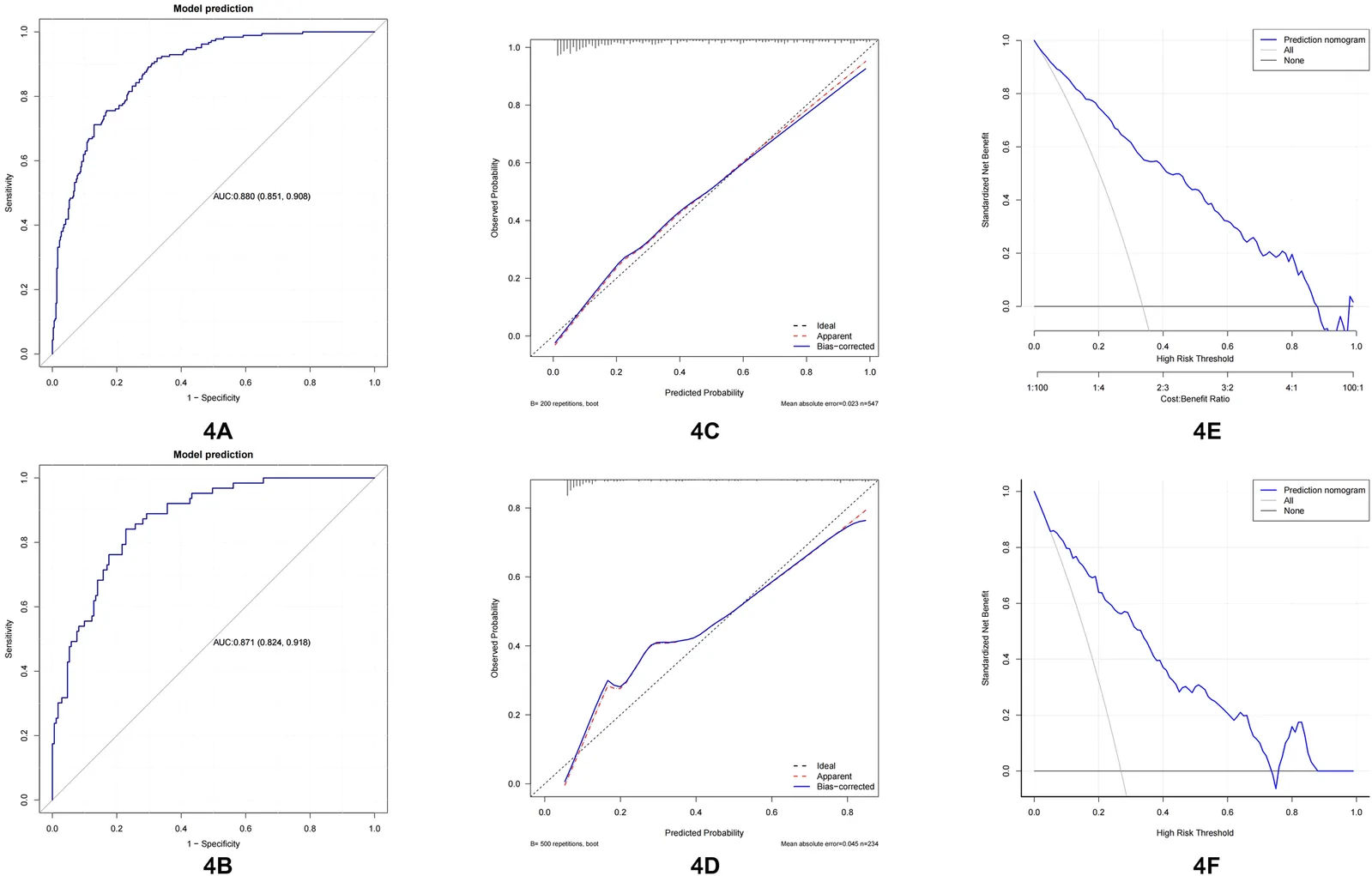
Performance and clinical utility evaluation of the nomogram in the training and validation cohorts. **(A)** ROC curve in the training cohort. The model demonstrated superior discriminative power with an AUC of 0.880 (95% CI: 0.851–0.908), indicating high predictive accuracy during the development phase. **(B)** ROC curve in the validation cohort. Robust performance was maintained in the independent dataset with an AUC of 0.871 (95% CI: 0.824–0.918), confirming the model's reliability and generalizability for individualized risk assessment. **(C)** Calibration curve in the training cohort. The curve demonstrates close concordance between the predicted and observed probabilities of sHAP. The Hosmer-Lemeshow test results (*χ*^2^ = 8.848, *P* = 0.355) indicate superior model fit and satisfactory calibration performance. **(D)** Calibration curve in the validation cohort. Consistent calibration performance was maintained in the independent dataset (*χ*^2^ = 10.551, *P* = 0.228), with high agreement between predicted and actual outcomes, confirming the model's robustness. **(E)** Decision curve analysis (DCA) in the training cohort. The nomogram yielded higher net benefit than both the “treat-all” and “treat-none” strategies across a wide range of threshold probabilities (2%–91%), indicating strong potential for clinical utility. **(F)** DCA in the validation cohort. The model maintained superior net benefit within threshold probabilities of 5%–73% in the independent dataset, confirming its robustness and clinical practical value for risk stratification and decision-making.

### Model calibration

The calibration curves showed close concordance between the predicted and observed probabilities of sHAP. The Hosmer-Lemeshow test indicated good model fit in both the training cohort (*χ*^2^ = 8.848, df = 8, *P* = 0.355) and the validation cohort (*χ*^2^ = 10.551, df = 8, *P* = 0.228), demonstrating satisfactory calibration performance (in Figures 4C,D).

### Clinical utility

Decision curve analysis was performed to evaluate the potential clinical usefulness of the nomogram. In the training cohort, the model demonstrated a higher net benefit than both the treat-all and treat-none strategies across a wide range of threshold probabilities (2%–91%). Similarly, in the validation cohort, the nomogram maintained superior net benefit within threshold probabilities ranging from 5% to 73%. These findings indicate that applying the model to guide clinical decision-making could improve risk stratification for sHAP compared with default management strategies (in Figures 4E,F).

## Discussion

In this retrospective cohort study, we developed and internally validated a bedside nomogram for predicting severe hospital-acquired pneumonia (sHAP) in patients with traumatic brain injury by integrating early-admission clinical variables with a semi-quantitative assessment of early chest CT findings. The model demonstrated robust discriminative performance, good calibration, and consistent clinical utility in both the training and validation cohorts. These findings suggest that quantitative evaluation of early pulmonary imaging features, when combined with key clinical indicators, may facilitate individualized risk stratification for sHAP in the neurocritical care setting. Importantly, this study highlights the potential value of incorporating early CT-based quantitative imaging markers into predictive models for pulmonary complications following TBI.

Hospital-acquired pneumonia remains one of the most frequent and severe complications following traumatic brain injury. Dysregulation of the brain-lung axis after TBI, characterized by sympathetic overactivation, systemic inflammation, and immune suppression, substantially compromises pulmonary host defense and predisposes patients to secondary infections (18). Severe hospital-acquired pneumonia is associated with prolonged mechanical ventilation, increased intensive care utilization, and excess mortality, highlighting the importance of early identification of patients at high risk (19). However, existing prediction approaches rely predominantly on clinical scores or laboratory biomarkers, which may be influenced by systemic inflammatory responses triggered by brain injury itself and often lack sufficient specificity for early bedside decision-making (20).

A key feature of the present model is the incorporation of early chest CT findings using a semi-quantitative scoring approach. Radiographic features of ground-glass opacities (GGO) and consolidations emerged as important imaging predictors of sHAP.

GGO represent areas of increased pulmonary attenuation with preservation of bronchial and vascular margins and are commonly considered to reflect early inflammatory or edematous changes within the lung parenchyma (21). After traumatic brain injury, systemic inflammatory activation and neurogenic pulmonary dysfunction may promote pulmonary vascular congestion and increased capillary permeability (22, 23), which may be radiologically manifested as ground-glass opacities on chest CT (24, 25). Previous studies have shown that the extent of GGO on CT correlates with pneumonia severity and adverse clinical outcomes. For example, Li MJ et al. reported that greater GGO involvement was significantly associated with increased disease severity and poorer prognosis (26).

In contrast, consolidations represent complete replacement of alveolar air by inflammatory exudate and generally indicate more advanced pulmonary involvement (27). The presence and extent of consolidations have been consistently associated with multilobar disease, greater pneumonia severity, and worse outcomes in patients with severe pulmonary infection (28). From a radiological perspective, consolidations may evolve from antecedent GGO as inflammatory exudation and alveolar filling progress. Therefore, the simultaneous presence of GGO and consolidations on early chest CT may reflect different stages and patterns of pulmonary injury.

Evaluating these two imaging patterns separately may allow the model to capture complementary information related to different phases of pulmonary involvement. Incorporating both features into the prediction model may therefore improve early risk stratification for sHAP beyond conventional clinical or laboratory parameters (29).

When selecting imaging variables for model construction, particular emphasis was placed on interpretability and reproducibility in routine clinical practice. Subtle airway-related features, such as air bronchograms and bronchial wall thickening, are often influenced by image quality, respiratory motion, and interobserver variability, which may limit their reliability in bedside assessment (30). In contrast, semi-quantitative evaluation of GGO and consolidations can be performed rapidly and consistently by clinicians with varying levels of experience, making these features suitable for inclusion in a pragmatic bedside prediction model.

In addition to imaging features, several clinical and laboratory variables were identified as independent predictors of sHAP. Advanced age and lower Glasgow Coma Scale scores reflect greater injury severity and impaired airway protection, both of which increase susceptibility to pulmonary infection (31). Mechanical ventilation, although often necessary in severe TBI, remains a well-recognized risk factor for nosocomial pneumonia (32). Elevated white blood cell counts may indicate systemic inflammatory activation, whereas lower lymphocyte counts may reflect post-traumatic immune suppression, a condition associated with increased infectious complications after TBI (31, 33). Furthermore, lower hemoglobin levels were inversely associated with sHAP risk, suggesting that impaired oxygen delivery and tissue hypoxia may further compromise pulmonary defense mechanisms (34).

By integrating these clinical, laboratory, and imaging variables, we constructed a concise nomogram that allows rapid estimation of individual sHAP risk at the bedside. The model demonstrated strong discrimination and calibration in both cohorts, and decision curve analysis indicated meaningful clinical benefit across a wide range of threshold probabilities. These findings suggest that the proposed nomogram may assist clinicians in identifying high-risk patients early after admission and support timely implementation of preventive and therapeutic strategies.

The clinical consequences of sHAP should also be considered when applying an early risk model. In patients with TBI, hospital-acquired pneumonia has been associated with longer acute hospitalization and poorer functional recovery (6). When pneumonia is suspected, clinical and microbiological reassessment, appropriate respiratory and hemodynamic support, and antimicrobial treatment informed by local susceptibility data and culture results are central to management (16). These published findings and management principles provide context for early risk identification; the present analysis did not evaluate the subsequent course of pneumonia, treatment response, or prognosis after sHAP.

## Limitations

Several limitations of this study should be acknowledged. First, the retrospective design and single-center setting may introduce selection bias and limit the generalizability of the findings. Second, although internal validation was performed using a separate validation cohort and bootstrap resampling, external validation in independent, multicenter populations is required to confirm the model's robustness and transportability. Third, chest CT features were assessed using a semi-quantitative visual scoring approach rather than automated quantitative or radiomics-based methods; while this choice enhances feasibility and reproducibility in routine clinical practice, it may limit the capture of more subtle imaging information. Fourth, the radiological scores were assessed by experienced physicians based on a predefined scoring system. Although a consensus approach was used to resolve discrepancies, formal interobserver reliability analysis was not performed, which may introduce potential measurement variability. Fifth, the model was constructed using variables collected early after admission and did not account for dynamic changes in clinical status or imaging findings during hospitalization, which may further influence the risk of sHAP. Sixth, antimicrobial exposure and neurosurgical procedures were not incorporated into the prespecified predictor set. Although treatment initiated after pneumonia onset and its eventual duration cannot be used for early prediction, antimicrobial exposure before the prediction time and operative management may influence subsequent risk. Their omission may limit the model's applicability across different treatment pathways. Future studies should record the indication and timing of antibiotic use, antibiotic agent and duration, and the type and timing of neurosurgical procedures, alongside the clinical course and outcomes after sHAP. Finally, although the nomogram incorporates readily available predictors, unmeasured confounders inherent to retrospective analyses cannot be fully excluded.

## Conclusion

In this study, we developed and internally validated a bedside nomogram for predicting severe hospital-acquired pneumonia in patients with traumatic brain injury by integrating early-admission clinical variables with a semi-quantitative assessment of chest CT findings. The model demonstrated robust discrimination, good calibration, and consistent clinical utility across both training and validation cohorts. By leveraging readily available imaging features together with key clinical and laboratory indicators, this nomogram enables early, individualized risk stratification and may assist clinicians in identifying patients at high risk for sHAP at an early stage of hospitalization. The proposed model represents a practical and interpretable tool that may support timely preventive and therapeutic decision-making in neurocritical care settings.

## Data Availability

The raw data supporting the conclusions of this article will be made available by the authors, without undue reservation.

## Supplementary material

Supplementary Figure S1 Representative CT Phenotypes and Semi-quantitative Assessment of Pulmonary Involvement in TBI Patients.

Supplementary Figure 1A A 56-year-old male was admitted 1 hour post-trauma following a motor vehicle accident. The axial CT scan reveals bilateral patchy ground-glass opacities (GGO) and areas of consolidation, predominantly distributed in the posterior segments. Based on the semi-quantitative scoring system utilized in this study, the GGO score for this patient was 2 (5%–25% involvement), and the consolidation score was 3 (26%–50% involvement).

Supplementary Figure 1B A 35-year-old male was admitted 2 hours post-trauma following a traumatic injury sustained at a construction site. The chest CT demonstrates bilateral ground-glass opacities (GGO) and focal consolidation, predominantly localized in the left lung. Based on the semi-quantitative scoring system utilized in this study, the GGO score for this patient was 1 (<5% involvement), and the consolidation score was 1 (<5% involvement).

Supplementary Figure 1C A 40-year-old male was admitted 2 hours post-trauma following a motor vehicle accident. The chest CT demonstrates bilateral patchy ground-glass opacities (GGO) and areas of consolidation, with GGO predominantly localized in the left lung. Based on the semi-quantitative scoring system utilized in this study, the GGO score for this patient was 2 (5%–25% involvement), and the consolidation score was 2 (5%–25% involvement).

Supplementary Figure 1D A 54-year-old male was admitted 2 hours post-trauma following a motor vehicle accident. The chest CT reveals extensive bilateral ground-glass opacities (GGO) and areas of consolidation, accompanied by massive pleural effusion on the right side and a small amount of pleural effusion on the left side. Excluding the pleural effusion, based on the semi-quantitative scoring system utilized in this study, the GGO score for this patient was 4 (51%–75% involvement), and the consolidation score was 2 (5%–25% involvement).

Supplementary Figure S2 Feature selection using LASSO regression for sHAP prediction. LASSO regression was employed to minimize multicollinearity and reduce the dimensionality of candidate predictors.

Supplementary Figure 2A illustrates the selection of the optimal tuning parameter through ten-fold cross-validation, displaying the trend of binomial deviance against the log-transformed parameter.

Supplementary Figure 2B presents the coefficient profiles and their shrinkage paths. This procedure identified ten essential variables with non-zero coefficients for the subsequent development of the multivariate predictive model.
