# Supplementary material for: Development and validation of a bedside nomogram for predicting severe hospital-acquired pneumonia in traumatic brain injury patients based on early semi-quantitative chest CT assessment
Source: Front Med (Lausanne). 2026 Oct 30;13:1926466. doi: 10.3389/fmed.2026.1926466 (PMC13760608; doi:10.3389/fmed.2026.1926466)
Supplement: Supplementary file 1 [file Datasheet5.pdf]

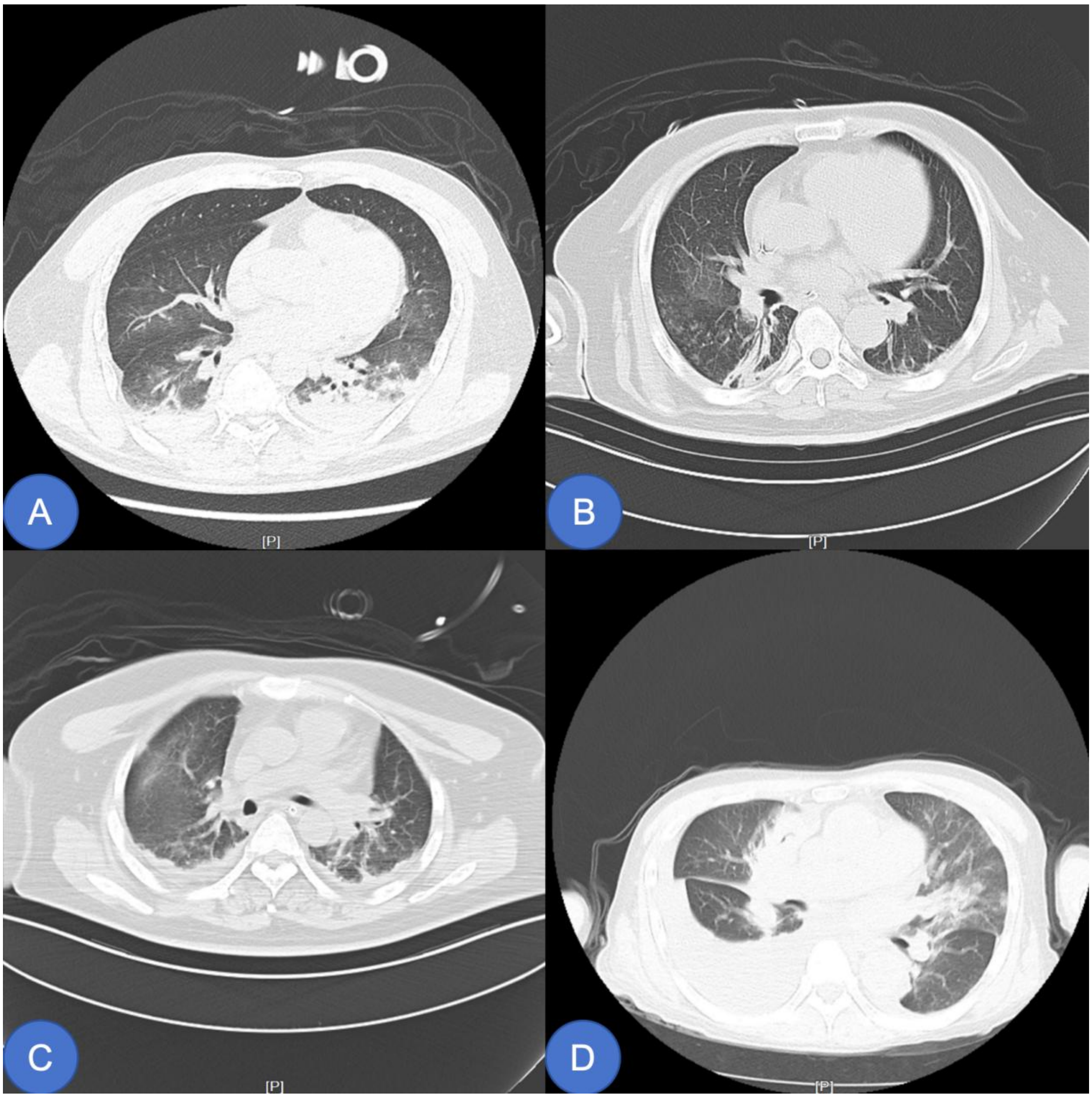

A
B
C
D
