# Supplementary material for: Development and validation of a bedside nomogram for predicting severe hospital-acquired pneumonia in traumatic brain injury patients based on early semi-quantitative chest CT assessment
Source: Front Med (Lausanne). 2026 Oct 30;13:1926466. doi: 10.3389/fmed.2026.1926466 (PMC13760608; doi:10.3389/fmed.2026.1926466)
Supplement: Supplementary file 2 [file Datasheet6.pdf]

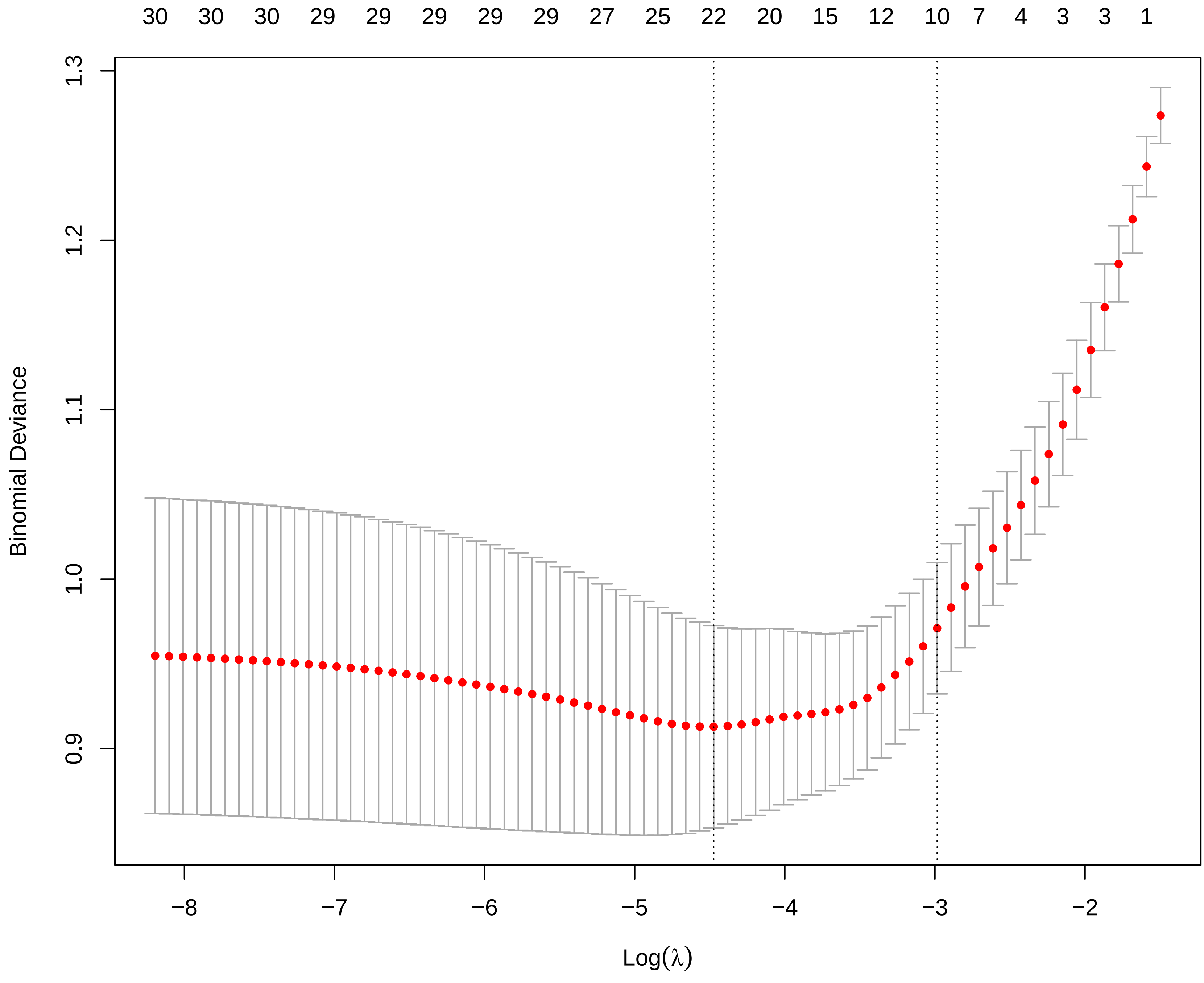

30 30 30 29 29 29 29 29 27 25 22 20 15 12 10 7 4 3 3 1
Binomial Deviance
1.3
1.2
1.1
1.0
0.9
−8 −7 −6 −5 −4 −3 −2
Log(λ)
