# Supplementary material for: Development and validation of a bedside nomogram for predicting severe hospital-acquired pneumonia in traumatic brain injury patients based on early semi-quantitative chest CT assessment
Source: Front Med (Lausanne). 2026 Oct 30;13:1926466. doi: 10.3389/fmed.2026.1926466 (PMC13760608; doi:10.3389/fmed.2026.1926466)
Supplement: Supplementary file 3 [file Datasheet7.pdf]

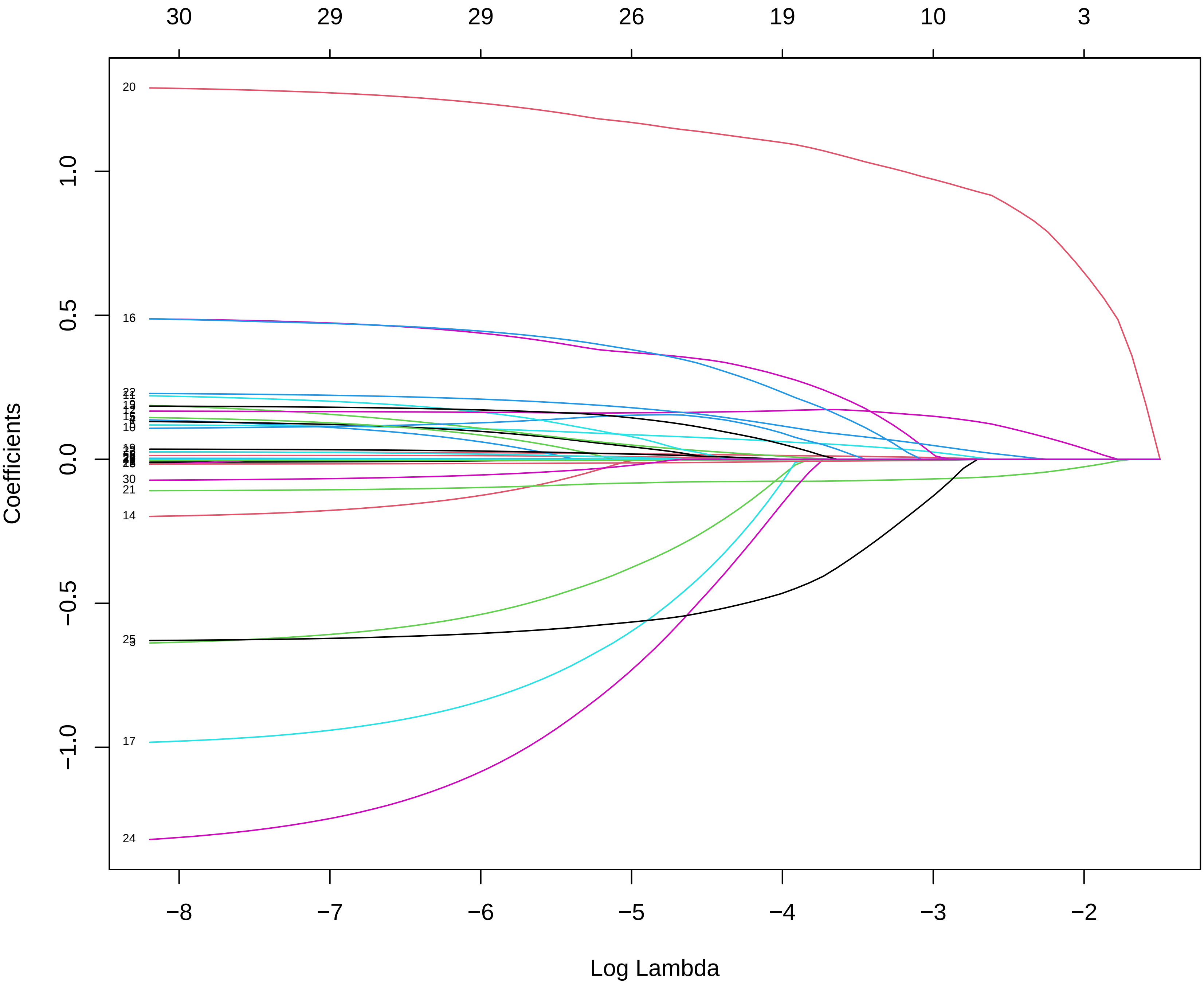

30
29
29
26
19
10
3
20
16
22
11
19
12
10
30
21
14
25
3
17
24
1.0
0.5
0.0
−0.5
−1.0
Coefficients
−8
−7
−6
−5
−4
−3
−2
Log Lambda
